# Accuracy of Preoperative Endoscopy in Determining Tumor Location Required for Surgical Planning for Esophagogastric Junction Cancer

**DOI:** 10.3390/jcm10153371

**Published:** 2021-07-29

**Authors:** Koichi Okumura, Yudai Hojo, Toshihiko Tomita, Tsutomu Kumamoto, Tatsuro Nakamura, Yasunori Kurahashi, Yoshinori Ishida, Seiichi Hirota, Hiroto Miwa, Hisashi Shinohara

**Affiliations:** 1 Division of Upper GI, Department of Gastroenterological Surgery, Hyogo College of Medicine, 1-1 Mukogawacho, Nishinomiya, Hyogo 663-8501, Japan; kokumura@kuhp.kyoto-u.ac.jp (K.O.); hojo@hyo-med.ac.jp (Y.H.); tkumamoto@hyo-med.ac.jp (T.K.); tnakamura@hyo-med.ac.jp (T.N.); kurahashi@hyo-med.ac.jp (Y.K.); ishida@hyo-med.ac.jp (Y.I.); 2 Division of Gastroenterology, Department of Internal Medicine, Hyogo College of Medicine, 1-1 Mukogawacho, Nishinomiya, Hyogo 663-8501, Japan; tomita@hyo-med.ac.jp (T.T.); miwahgi@hyo-med.ac.jp (H.M.); 3 Department of Surgical Pathology, Hyogo College of Medicine, 1-1 Mukogawacho, Nishinomiya, Hyogo 663-8501, Japan; hiros@hyo-med.ac.jp

**Keywords:** esophagogastric junction cancer, esophagogastric junction, narrow band imaging, Barrett’s esophagus

## Abstract

Purpose: The surgical strategy for esophagogastric junction (EGJ) cancer depends on the tumor location as measured relative to the EGJ line. The purpose of this study was to clarify the accuracy of diagnostic endoscopy in different clinicopathological backgrounds. Methods: Subjects were 74 consecutive patients with abdominal esophagus to upper gastric cancer who underwent surgical resection. Image-enhanced endoscopy with narrow-band imaging (NBI) was used to determine the EGJ line, prioritizing the presence of palisade vessels, followed by the upper end of gastric folds, as a landmark. The relative positional relationship between the tumor epicenter and the EGJ line was classified into six categories, and the agreement between endoscopic and pathologic diagnoses was examined to evaluate prediction accuracy. Results: The concordance rate of 69 eligible cases was 87% with a kappa coefficient (*K*) of 0.81. The palisade vessels were observed in 62/69 patients (89.9%). Of the 37 pathological EGJ cancers centered within 2 cm above and below the EGJ line, Barrett’s esophagus was found to be a significant risk factor for discordance (risk ratio, 4.40; *p* = 0.042); the concordance rate of 60% (*K* = 0.50) in the Barrett’s esophagus group was lower than the rate of 91% (*K* = 0.84) in the non-Barrett’s esophagus group. In five of six discordant cases, the EGJ line was estimated to be proximal to the actual line. Conclusion: Diagnostic endoscopy is beneficial for estimating the location of EGJ cancer, with a risk of underestimating esophageal invasion length in patients with Barrett’s esophagus.

## 1. Introduction

Esophagogastric junction (EGJ) malignancies are becoming increasingly common and account for about one-third of all upper digestive-tract cancers in Western countries [1,2,3,4]. In East Asian countries, including Japan, cases of EGJ adenocarcinoma are likewise increasing, probably due to *Helicobacter pylori* eradication efforts, smoking habits, obesity, and increased incidence of gastroesophageal reflux disease [5,6,7,8]. There are two major classifications for EGJ cancer: Nishi’s classification and Siewert’s classification. Nishi’s classification defines tumors according to the location of their epicenter within 2 cm above and below the EGJ line regardless of their histological subtype [9,10]. They are comparable with Siewert type II EGJ cancer, where tumors are localized within 1 cm above and 2 cm below the EGJ line and are referred to as “true carcinoma of the cardia” [11,12,13]. In both classifications, the EGJ line is an important reference point for surgery.

When planning the surgical strategy for EGJ cancer, both the location of the epicenter of the tumor and the extent of the tumor will influence whether gastrectomy or esophagectomy is performed [14]. The distance of esophageal invasion from the EGJ line should also be considered when planning surgery, given that several retrospective and recent prospective studies have demonstrated its association with the frequency of mediastinal lymph node metastasis [15,16,17,18,19]. However, determining the EGJ line precisely before surgery is often challenging, especially when tumors are bulky or are concomitant with Barrett’s esophagus or esophageal hiatal hernia [20,21].

Anatomically, the EGJ line refers to the muscular border of the esophagus and stomach, where the lower edge of the lower esophageal sphincter (LES) coincides with the EGJ line. Endoscopically, the EGJ is defined by the upper end of the gastric folds [22], although the criteria for determining this location are not specific. In Japan, the palisade vessels are used as a landmark to distinguish the EGJ [23,24,25]. Although these longitudinal vessels present in the mucosal layer of the LES are consistently located, they are considered difficult to detect in Western populations [26].

Recent advances in endoscopic imaging have afforded greatly improved resolution and magnification. Image-enhanced endoscopy using narrow band imaging (NBI) can now help visualize the microvasculature on the mucosal surface, which allows the palisade vessels to be visualized more readily. Studies in Japanese [27] and Western [28] populations have suggested that these vessels can be a landmark for endoscopic recognition of the EGJ.

The purpose of this study was to clarify the accuracy and reliability of endoscopic diagnosis in estimating the location of the EGJ line, which is necessary for planning the surgical strategy in patients with EGJ cancer.

## 2. Materials and Methods

### 2.1. Patients

This retrospective, single-center, observational study investigated a total of 74 consecutive patients with the tumor epicenter located between the abdominal esophagus and the upper gastric fundus who underwent open or laparoscopic surgery at the Hyogo College of Medicine from January 2016 to December 2018. Exclusion criteria were declining to participate and receiving endoscopic submucosal dissection (ESD) or endoscopic mucosal resection as preoperative treatment. The operative procedures performed were transthoracic esophagectomy with lymphadenectomy, transhiatal esophagectomy with lymphadenectomy, total gastrectomy with lymphadenectomy, or proximal gastrectomy with lymphadenectomy, as determined preoperatively by our institutional cancer board and described in detail elsewhere [14].

### 2.2. Endoscopic Examination

All patients underwent image-enhanced endoscopy (endoscopes: GIF-Q260, -H260, -H260Z, -H290, -HQ290, or -H290Z; Olympus Medical Systems, Tokyo, Japan) with an electronic endoscopic system (Elite CV290; Olympus Medical Systems). Endoscopy was performed by a single highly experienced endoscopist (T.T.) certified by the Japan Gastroenterological Endoscopy Society. Patients lay on their left side, with the upper body slightly elevated under conscious sedation. After air was suctioned from the stomach, the examinee inhaled deeply with the lower esophagus adequately stretched and endoscopic observation of the palisade vessels was performed using white light imaging (WLI) first, followed by NBI for visualizing the palisade vessels if they were undetectable by WLI [27,28]. All NBI images of palisade vessels were obtained in normal mode with confirmation under magnification. During observation, we adequately washed the esophageal mucosa with dimethicone solution. The location of the tumor epicenter was defined as the mid-point on the longitudinal axis, and the distance of tumor invasion from the EGJ line was estimated based on the endoscope’s diameter or measured directly if the length was within 1 or 2 cm.

### 2.3. Image Assessment

**Figure 1:**
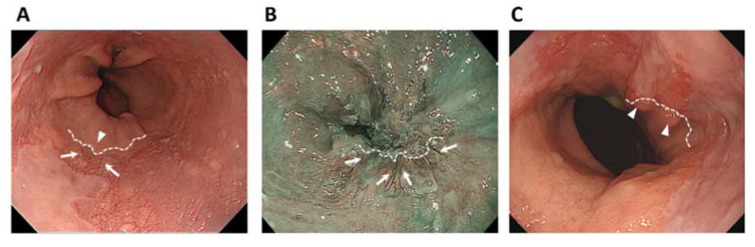
Endoscopic images from which the esophagogastric junction (EGJ) line was estimated. (**A**) A representative white light image of a non-cancer patient with Barrett’s esophagus. The EGJ line was estimated (broken line) where the lower end of the palisade vessels (arrows) and the upper end of the gastric folds (arrowheads) are almost coincident. (**B**) A narrow-band image of an EGJ cancer patient. The EGJ line was estimated from the lower end of the palisade vessels (arrows). (**C**) A white light image of an EGJ cancer patient. The EGJ line was estimated from the upper end of the gastric folds because the palisade vessels could not be visualized.

**Figure 2:**
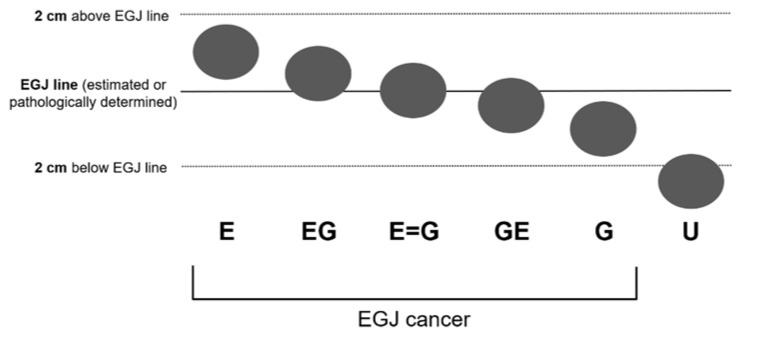
Classification of the epicenter of the tumor determined in relation to the distance from the esophagogastric junction. EGJ—esophagogastric junction.

Prior to surgery, two endoscopists (T.T. and H.M.) and seven surgeons (K.O., Y.H., T.K., T.N., Y.K., Y.I., and H.S.) who participated in the cancer board reviewed the endoscopic images of each patient to estimate the location of the EGJ line, which was determined first relative to the lower end of the palisade vessels and second relative to the upper end of the longitudinal folds of the stomach (Figure 1). Then, the location of the tumor epicenter relative to the EGJ line was classified into six categories based on the 11th edition of the Japanese Classification of Esophageal Cancer [10], as shown in Figure 2. These six categories are E, EG, E = G, GE, G, and U.

### 2.4. Evaluation of Accuracy

After surgery, the surgical specimen was immediately submitted in 10% neutral buffered formalin for pathological analysis, and the fixation time was approximately 24 h. The positional relationship was re-categorized based on the pathologic diagnosis of the surgical specimen. The presence of Barrett’s esophagus, which was defined as columnar metaplasia of the esophagus, was histologically examined in accordance with the diagnostic criteria [29] by an expert pathologist (S.H.). The accuracy of the estimated location in relation to the EGJ line was evaluated by comparing the tumor location category before and after the operation in each case and calculating the concordance rate.

### 2.5. Statistics

Continuous variables are expressed as the mean (standard deviation) or median (range). Categorical variables are expressed as number of cases, indicating prevalence. Tumor location or esophageal involvement was determined by the pathologic diagnosis as the gold standard, and concordance was calculated using the kappa coefficient (*k*), which takes into account the possibility of the agreement occurring by chance [30]. Concordance rate was defined as the rate of perfect concurrence of the locations of the tumor epicenter determined before and after surgery. The risk factors for positional discordance were evaluated using Fisher’s exact test. *p* values below 0.05 were considered significant in all statistical analyses. Statistical analyses were performed using JMP Statistics software version 13.0 (SAS Institute Inc. Cary, NC, USA).

### 2.6. Ethics

This study was approved by the Ethics Committee of Hyogo College of Medicine (Approval No. 3142). The study complied with the Declaration of Helsinki and the Ethical Guidelines for Medical and Health Research Involving Human Subjects stipulated by the Japanese national government for the protection of patient anonymity.

## 3. Results

**Figure 3:**
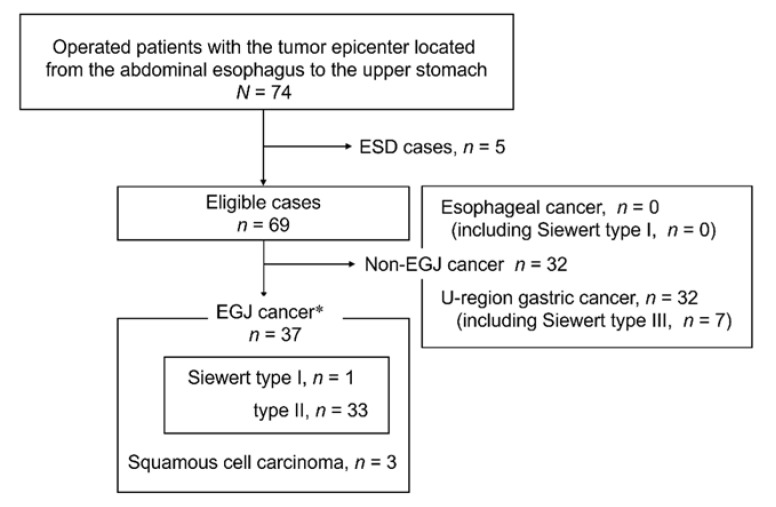
Flow chart of this study. * According to Nishi’s classification. ESD—endoscopic submucosal resection; EGJ—esophagogastric junction.

All 74 consecutive patients underwent surgical resection with curative intent for histologically confirmed adenocarcinoma or squamous cell carcinoma (SCC) whose epicenter was located between the abdominal esophagus and the upper gastric fundus. Five patients who underwent preoperative endoscopic treatment were excluded, leaving 69 patients for the analysis. Of these 69 patients, 37 had a pathological diagnosis of EGJ cancer based on Nishi’s classification (Figure 3). The EGJ line could be determined with the palisade vessels as a landmark in 62 of the patients and with the upper gastric folds in the remaining seven patients.

The categories of tumor location relative to the EGJ line estimated before surgery and pathologically confirmed after surgery were collated for each of 69 patients and the results are shown in Table 1. The concordance for EG, E = G, GE, G, and U was 1.00, 0.82, 0.80, 0.75, and 0.91, respectively, and *k* was calculated as 0.81. Overall, concordance was seen in 60 of the 69 patients (87%). In the remaining 9 discordant cases, the preoperatively estimated category was more proximal than the pathologically confirmed category in three cases and more distal in six cases. There were three cases that were preoperatively categorized as U-region gastric cancer but pathologically confirmed to be EGJ cancer.

Table 2 shows the clinicopathological backgrounds of the 37 patients with pathologically diagnosed EGJ cancer. Mean age was 67 years and the male-to-female ratio was 2.7:1. Mean tumor size was 40 mm, with 16 tumors larger than that, and maximum diameter was 79 mm. Three patients were diagnosed with SCC, and one patient with adenocarcinoma was found to have achieved complete remission by postoperative pathological diagnosis after neoadjuvant chemotherapy. Concomitant Barrett’s esophagus was evident in 15 patients (41%) and esophageal hiatus hernia in 16 patients (43%).

Table 3 shows the results of the analysis of potential risk factors that could cause discordance in the diagnosis of EGJ cancer. Concomitant Barrett’s esophagus was the only significant factor (risk ratio 4.40, 95% confidence interval, 1.022–18.9; *p* = 0.042), not concomitant esophageal hiatus hernia or longitudinal tumor axis >4 cm. Table 4 shows the results of collation of the preoperatively estimated and pathologically confirmed categories in each of the 37 patients with EGJ cancer. The concordance for EG, E = G, GE, and G in the 15 patients with concomitant Barrett’s esophagus was 1.00, 0.67, 0.33, and 1.00, respectively. For the three patients with pathological EGJ cancer that were preoperatively categorized as U (GE in 2 and G in 1), the concordance rate was only 60% (*k* = 0.50). This was markedly lower than for the 22 non-Barrett’s esophagus patients, where concordance for EG, E = G, GE, and G was 1.00, 0.88, 1.00, and 0.50, respectively, and the concordance rate reached 91% (*k* = 0.84). Notably, in five of the six discordant cases with concomitant Barrett’s esophagus, the preoperative estimated category was more distal than the pathologically confirmed category, meaning that the EGJ line was estimated proximal to the actual line. The palisade vessels could not be used as a landmark for the EGJ line in all discordant cases.

## 4. Discussion

Information about tumor location and esophageal involvement are essential for planning the optimal resection procedure and extent of lymph node dissection for EGJ cancer, but it can be difficult to precisely determine the EGJ line as a reference point. In this study, by comparing against the postoperative (pathologically confirmed) diagnosis, we found that preoperative image-enhanced endoscopy using the palisade vessels as the landmark is highly reliable at estimating the location of the EGJ line, with a high concordance rate of 87% and *k* value of 0.81. Among the various clinicopathological backgrounds examined, concomitant Barrett’s esophagus was a significant risk factor for discordance.

The EGJ line refers to the muscular border of the esophagus and stomach. Its actual location can be known only on pathological diagnosis of the surgical specimen and cannot be seen directly through the mucosa on the lumen side [23,24] nor marked in preoperative endoscopy. Therefore, it is methodologically difficult to evaluate how well our estimations match reality. In this study, we classified the relative positional relationship between the tumor epicenter and the estimated EGJ line into six categories and then compared the location estimated on preoperative endoscopy with that confirmed pathologically. This method was helpful for broadly diagnosing whether the tumor epicenter was on the esophageal side or gastric side and could be easily applied to Siewert’s classification [11,12].

Western guidelines define the EGJ line as the upper end of the gastric folds [22]. This landmark is easy to identify, but its position may vary due to factors such as air insufflation or deep inspiration [26], which may in turn influence the preoperative assessment of EGJ cancer location. Grotenhuis et al. reported an overall accuracy of 70% in predicting tumor location according to the Siewert classification, which takes the proximal extent of the gastric folds as the EGJ line, and they concluded that the usefulness of endoscopic prediction is limited [20]. Pedrazzani reported that the accuracy of endoscopic ultrasonography in determining Siewert type was 72.5%, and the specificity in distinguishing between type II and type III tumors was very low (44%) [21]. The Japanese endoscopy guidelines define the EGJ line as the distal extent of the palisade vessels present in the mucosal layer within the LES [10]. Although the palisade vessels are a more consistent EGJ landmark than the upper gastric folds, they have been described as faint and not always clearly visible in Western patients [26]. In this study, we found that current endoscopic imaging techniques can detect the palisade vessels in most EGJ cancer patients with a variety of clinicopathological backgrounds. Endoscopic observation with the patient under shallow sedation and inhaling deeply with the lower esophagus adequately stretched may also facilitate detection of the palisade vessels.

Barrett’s esophagus is a premalignant condition often associated with EGJ cancer and responsible for its development [1]. Endoscopic diagnosis that focuses on fine mucosal patterns and capillary patterns using an NBI magnifying endoscope is already established [31,32,33]. Defining the EGJ line as the distal margin is important for estimating the segment that extends to the lower esophagus. Although Western endoscopists have not used the palisade vessels as a landmark for the reasons mentioned above, a recent report from the Netherlands showed that these vessels were in fact detectable in most Western patients with Barrett’s esophagus (92%) [28]. In the present study, Barrett’s esophagus was the main factor for discordance, mainly involving estimations proximal to the actual EGJ line. Tumors existing in the EGJ area may have caused concomitant esophagitis, obscuring the palisade vessels. Indeed, in all discordant cases, the palisade vessels could not be observed and the gastric folds were used as an alternative landmark for the EGJ line. Schölvinck et al. reported that the lower ends of the palisade vessels are located more distal than the upper ends of the gastric folds [28]. Using the gastric folds as a landmark in the present study may have led to the EGJ line being estimated more proximally. It is also possible that in tumors misdiagnosed as upper gastric cancer, even the gastric folds may not have been useful as an alternative landmark for the EGJ line. Imprecise estimations of the EGJ line to the proximal side could carry the risk of underestimating the esophageal invasion and in turn affect surgical planning, including the extent of lymph node dissection [14,18].

This study had some limitations. This was a retrospective observational study involving a small number of patients at a single institution where endoscopic procedures and techniques could be readily controlled. In addition, because of the sample size limitations, only univariate analysis was conducted. In our analysis, tumor size >4 cm was not identified as a risk factor for discordance, but bulky tumors that grow circumferentially will make it difficult to estimate the EGJ line. Although endoscopy can be a useful diagnostic method, naturally other imaging modalities such as computed tomography are necessary for comprehensive preoperative diagnosis and surgical planning.

## 5. Conclusions

Accurately estimating the location of the EGJ line is important in planning the optimal operative procedure for tumors that have developed close to it. Preoperative diagnostic endoscopy that uses the palisade vessels first as a landmark corresponds to the postoperative pathological diagnosis in almost 90% of cases. Concomitant Barrett’s esophagus is a risk factor for discordance, estimating the EGJ line more proximal than it actually is, which could lead to underestimation of esophageal invasion.

## Figures and Tables

**Table 1:** Collation of the preoperatively estimated and pathologically confirmed tumor categories for 69 eligible patients with a tumor epicenter located between the abdominal esophagus and the upper gastric fundus.

		Pathological Category							
		**E ^1^**	**EG ^1^**	**E = G ^1^**	**GE ^1^**	**G ^1^**	**U**		**Concordance**
								**Total**	
Preoperative category	E ^1^	0^2^	0	0	0	0	0	0	NA
	EG ^1^	0	5 ^2^	0	0	0	0	5	1.00
	E = G ^1^	0	0	9 ^2^	1 ^3^	1 ^3^	0	11	0.82
	GE ^1^	0	1 ^4^	1 ^4^	12 ^2^	0	1	15	0.80
	G^1^	0	0	0	1 ^4^	3 ^2^	0	4	0.75
	U	0	0	0	1 ^4^	2 ^4^	31 ^2^	34	0.91
	Total	0	6	10	15	6	32	69	

^1^ EGJ cancer according to the Nishi’s classification; ^2^ concordant cases; ^3^ discordant cases where the preoperatively estimated category was more proximal than the pathologically confirmed category; ^4^ discordant cases where the preoperatively estimated category was more distal to the pathologically confirmed category. EGJ—esophagogastric junction; NA—not available.

**Table 2:** Characteristics of patients with pathologically diagnosed EGJ cancer (*n* = 37).

	*n* (%)
Age (years) _1_	67 ± 13
Sex (M/F)	27/10
BMI	21.8 ± 3.0
Tumor size (mm, major axis) ^1^	40 ± 19
Barrett’s esophagus	15 (41)
Esophageal hiatus hernia	16 (43)
Histology	
Squamous cell carcinoma	3 (8)
Adenocarcinoma	33 (89)
pap, tub1	8 (22)
tub2	17 (46)
por, muc	8 (22)
cT	
1b	11 (30)
2	6 (16)
3	14 (38)
4a	6 (16)
cN	
0	18 (49)
1	7 (19)
2	8 (22)
3	4 (11)
Operative procedure	
Total Gastrectomy	19 (51)
Proximal Gastrectomy	10 (27)
Esophagectomy	8 (22)
Operative time (min) ^2^	450 (202–751)
Blood loss (mL) ^2^	80 (0–850)

^1^ Mean ± standard deviation; ^2^ median (range); BMI—body mass index; pap—papillary adenocarcinoma; tub1—well differentiated adenocarcinoma; tub2—moderately differentiated adenocarcinoma; por—poorly differentiated adenocarcinoma; muc—mucinous adenocarcinoma.

**Table 3:** Potential risk factors for discordance among the classification categories of esophagogastric junction cancer.

Factor	RR	(95%CI)	*p*-Value
Sex M vs. F	2.59	(0.363–18.5)	0.404
Age ≥75 vs. <75	3.47	(0.990–12.2)	0.080
Barrett’s esophagus	4.40	(1.022–18.9)	0.042
Esophageal hiatus hernia	2.19	(0.611–7.83)	0.254
Major axis of tumor ≥4 vs. <4 cm	1.60	(0.423–6.06)	0.674

CI—confidence interval; RR—risk ratio.

**Table 4:** Collation of preoperatively estimated and pathologically confirmed tumor categories in 37 EGJ cancer patients with or without concomitant Barrett’s esophagus.

		Pathological Category						
		**E ^1^**	**EG ^1^**	**E ^1^ = G**	**GE ^1^**	**G ^1^**		**Predictive Value**
							**Total**	
**With Barrett’s Esophagus**								
Preoperative category	E ^1^	0^2^	0	0	0	0	0	NA
	EG ^1^	0	4^2^	0	0	0	4	1.00
	E = G ^1^	0	0	2 ^2^	0	1^3^	3	0.67
	GE ^1^	0	1^4^	1 ^4^	1^2^	0	3	0.33
	G^1^	0	0	0	0 ^4^	2 ^2^	2	1.00
	U	0	0	0	1 ^4^	2 ^4^	3	0.00
	Total	0	5	3	2	5	15	
**Without Barrett’s Esophagus**								
Preoperative category	E ^1^	0 ^2^	0	0	0	0	0	NA
	EG ^1^	0	1 ^2^	0	0	0	1	1.00
	E = G ^1^	0	0	7 ^2^	1 ^3^	0	8	0.88
	GE ^1^	0	0	0	11 ^2^	0	11	1.00
	G ^1^	0	0	0	1 ^4^	1 ^2^	2	0.50
	U	0	0	0	0	0	0	NA
	Total	0	1	7	13	1	22	

^1^ EGJ cancer according to the Nishi’s classification; ^2^ concordant cases; ^3^ discordant cases where the preoperatively estimated category was more proximal than the pathologically confirmed category; ^4^ discordant cases where the preoperatively estimated category was more distal to the pathologically confirmed category. EGJ—esophagogastric junction; NA—not available.

## Data Availability

The data presented in this study are available on request from the corresponding author. The data are not publicly available due to ethical restrictions.
